# Suprasternal approach for insertion of Impella 5.5 into the proximal right subclavian artery

**DOI:** 10.1007/s12055-024-01699-5

**Published:** 2024-02-12

**Authors:** Jay A. Patel, Zubair A. Hashmi

**Affiliations:** Department of Cardiothoracic Surgery, Pauley Heart Center, Virginia Commonwealth University, Richmond, VA USA

**Keywords:** Impella 5.5, Cardiogenic shock, Subclavian Impella, Innominate Impella

## Abstract

The Impella 5.5 (Abiomed) is a percutaneous, temporary left ventricular assist device (LVAD) that serves as an important method of treatment of acute cardiogenic shock refractory to medical management. The Impella 5.5 and 5.0 are commonly inserted through the right axillary artery; however, this may be limited by inadequate vessel diameter to accommodate the Impella and inadequate vessel quality. A central approach to Impella 5.5 incision has been described in the pediatric population, particularly via the innominate artery through a suprasternal and/or neck incision, with success. As an alternative to axillary Impella placement, we propose the usage of a limited suprasternal incision for Impella 5.5 insertion in the adult population, either through the proximal right subclavian artery or the distal innominate artery. This may offer multiple advantages, such as increased vessel diameter and quality of more proximal vessels, avoidance of partial sternotomy, avoidance of a second infraclavicular wound site if the patient progresses to require LVAD or transplant, avoidance of lymphatic and nerve injury through the axillary exposure, ease of manipulation for repositioning, and patient rehabilitation. Potential limitations include difficulty due to body habitus, potential risk of stroke with the innominate approach, and wound complications. A central approach is a reasonable alternative to axillary Impella placement in patients with inadequate axillary artery caliber, defined as less than 6–7 mm diameter, poor artery quality to accommodate anastomosis, and small body habitus, allowing for ease of exposure.

## Introduction

The Impella 5.5 (Abiomed, Danvers, MA, USA) is a percutaneously inserted transvalvular, micro-axial temporary left ventricular assist device (LVAD) that functions to unload the left ventricle in cardiogenic shock and provide circulatory support, allowing for myocardial recovery [1]. The Impella 5.5 is the most recent percutaneous LVAD and is intended for use for up to 14 days, delivers up to a maximum of 5.5 l of blood per minute, and is typically inserted through an axillary artery cutdown, a location which allows for patient rehabilitation [1]. Indications for use include (1) cardiogenic shock after myocardial infarct, myocarditis with left ventricular failure, or post-cardiotomy shock refractory to maximal medical management which includes fluids, pressors, and intra-aortic balloon pump (IABP); (2) support during high-risk percutaneous coronary intervention (PCI) or during coronary artery bypass or valve replacement; (3) treatment of ventricular distension and pulmonary edema on patients on extracorporeal membrane oxygenation (ECMO), and; (4) treatment of severe hemolysis related to Impella CP support [2]. Contraindications to use include (1) left ventricle mural thrombus; (2) presence of mechanical aortic valve, severe aortic stenosis, moderate to severe aortic insufficiency, or aortic valve calcification; (3) severe arterial disease preventing placement; (4) atrial or ventricular septal defect; (5) severe right heart failure; (6) left ventricular rupture, tamponade, or combined cardiorespiratory failure [1]. Insertion typically involves utilizing the axillary artery, which is exposed through an infraclavicular incision, suturing a graft in an end-to-side fashion to the artery, for the retrograde endovascular delivery of the Impella device across the aortic valve, into the left ventricle [2]. In this report, we describe the use of using the proximal right subclavian artery through a suprasternal incision for the insertion of the Impella 5.5, as a reasonable alternative in specific patient populations, to the commonly used axillary artery.

## Technique

In our experience, we prefer to perform Impella placement in the standard right axillary position; however, in patients with smaller body habitus with inadequate axillary artery vessel caliber or prohibitive vascular disease to accommodate the Impella, our preference is to place the Impella more centrally into the proximal subclavian or distal innominate artery. A computed tomographic angiography (CTA) of the chest can be obtained in patients suspected of having small vessel caliber secondary to small body habitus, to assess the adequacy of vessel diameter to accommodate the Impella. In our overall experience, we have resorted to this alternate approach very rarely, in a total of eight patients, while the large majority have been performed in the standard right axillary position. Of these eight patients, seven were performed for small vessel caliber (one of which was an aborted supraclavicular approach for inadequate proximal vessel caliber with variant carotid anatomy), and one was performed in the setting of a left-sided implantable cardioverter-defibrillator (ICD) that was re-sited to the right, thus necessitating a central approach to avoid a re-operative field.

**Fig. 1:**
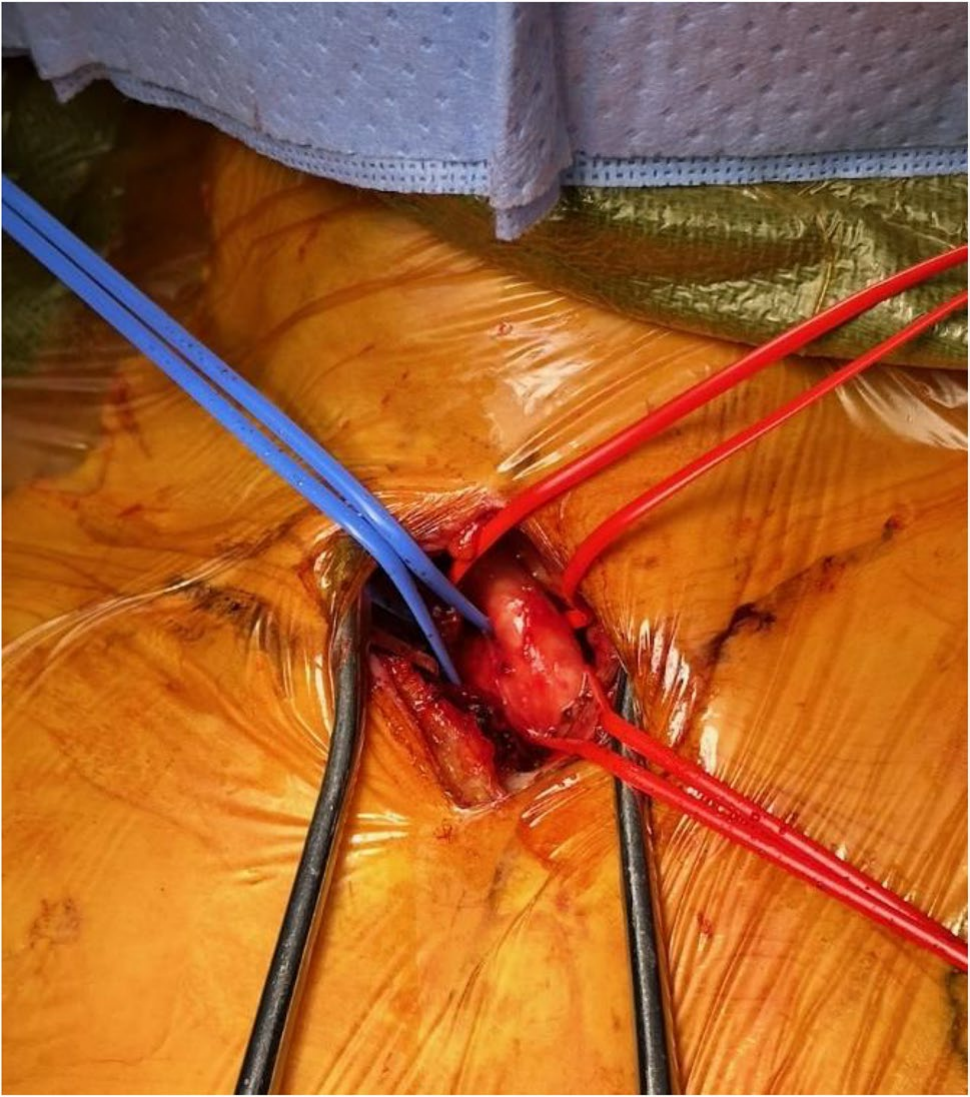
Suprasternal exposure of the proximal right subclavian artery (1), distal innominate artery (2), and proximal right common carotid artery (3)

**Fig. 2:**
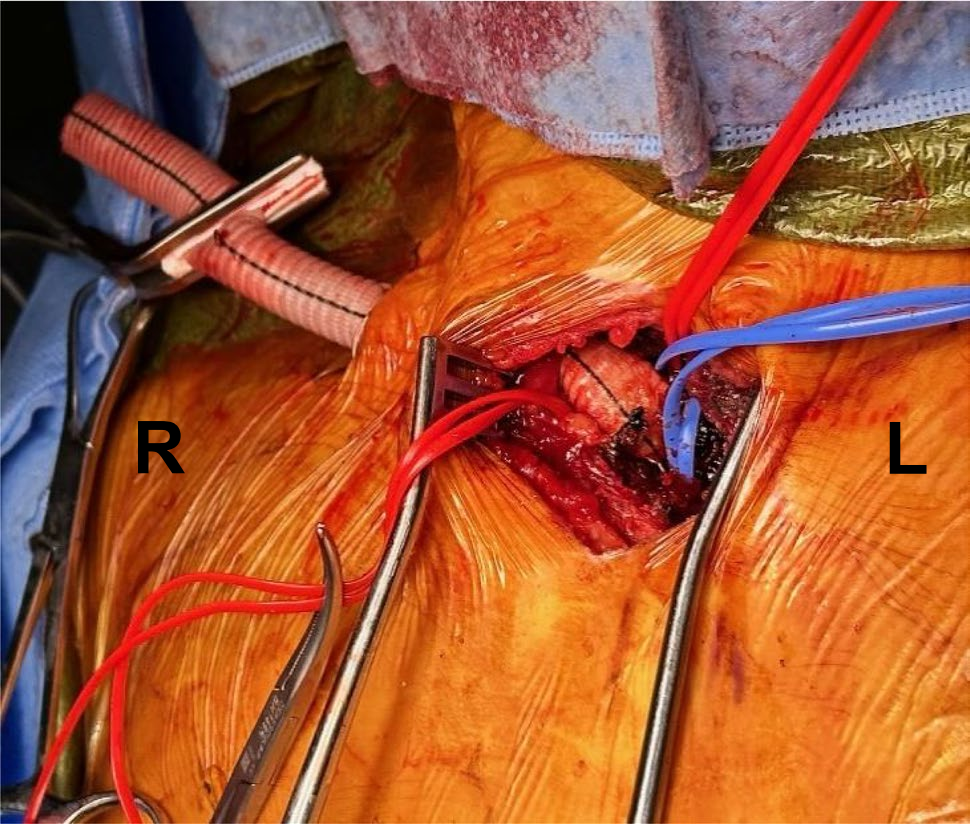
End-to-side graft sutured to proximal subclavian artery tunneled through a supraclavicular incision, ready for insertion of the Impella 5.5

Our standard approach in a patient with small vessel caliber begins with adequate pre-operative preparation and patient positioning with neck extension and a shoulder roll to expand the neck space. The choice of incision is made based on the best approach to the target vessel, which is aided by CTA and can involve a vertical suprasternal incision with supraclavicular extension or vice versa. For the suprasternal approach, a vertical incision is made at the sternal notch. The dissection is carried through the subcutaneous tissues, and the strap muscles may need to be mobilized to provide exposure to the vessels. Sternotomy is not required through this incision, which provides exposure to the distal innominate artery, and proximal right subclavian and proximal common carotid arteries. With appropriate patient selection, we have not needed to convert to an upper partial sternotomy for exposure. Control with vessel loops is obtained of the arteries (Fig. 1), and heparin is given to achieve an activated clotting time (ACT) of at least 250 s. Vessel loops along the proximal right subclavian artery are snared, and a longitudinal arteriotomy is made to accommodate a 10-mm synthetic graft, which is anastomosed end-to-side using running 5-0 polypropylene suture. This approach does not compromise the carotid artery. The vessel loops are loosened, and the graft is de-aired and back-bled, the graft is then tunneled through the right supraclavicular space, and a sheath is attached for subsequent Impella 5.5 insertion (Fig. 2).

## Discussion

An axillary or subclavian approach has long been preferred for Impella placement as it allows for percutaneous left ventricular support while allowing for patient rehabilitation and reflects the majority of our experience with the Impella. In our overall experience performing the alternative, central placement of the Impella graft through a suprasternal incision in a very limited patient population, we have not had complications related to the graft location or placement itself. Complications related to the Impella device placement were not deemed relevant to our paper as Impella complications occur irrespective of the location chosen for the entry graft.

Implantation of the Impella 5.5 is feasible through small arterial vessels, as described by Shugh et al., who implanted an Impella 5.5 into the axillary artery of an 11-year-old patient, with a subclavian artery size of 4.8 × 5.1 mm, which is less than the recommended 7 mm for adult implantation, with good outcomes [3]. An axillary artery diameter of at least 6 mm is required for Impella insertion through the axillary artery [4]. Limitations due to small arterial size may be overcome by using a more proximal, and larger caliber subclavian artery, and if technically feasible, the innominate artery. However, the innominate artery approach necessitates a side-biting clamp for anastomosis as this artery cannot be fully clamped given the risk of cerebral ischemia. Additionally, placement of an adequately sized side-biting clamp on the innominate artery can be difficult through the small access incision and limitations by the sternal notch. In a report by Bouhout et al., a trans-innominate Impella 5.5 was implanted through a partial sternotomy in a 14-year-old patient. An upper partial sternotomy was performed down through the right third intercostal space, the innominate vein was retracted inferiorly, and the innominate artery was exposed without entering the pericardium [4]. After anticoagulation, a 10-mm graft was anastomosed end-to-side to the artery using 5-0 polypropylene and tunneled out through the supraclavicular space, with subsequent placement of the Impella 5.5 [4]. We propose the option of the avoidance of a partial sternotomy through a suprasternal incision for exposure of the proximal subclavian artery or innominate artery. A central approach may be feasible in both adults and the pediatric population [4] and may also avoid problems related to small or diseased peripheral arteries. Also, central entry of the Impella 5.5 allows for easier manipulation and repositioning given its more direct path to the left ventricle, versus an axillary Impella 5.5, which has to go through multiple turns to reach the left ventricle, thus limiting the ease of manipulation [5].

A similar approach to innominate exposure was described by Harvey et al. in a 17-year-old patient. An oblique incision was made from the sternal notch extending 3 cm onto the anterior border of the sternocleidomastoid, and with lateral retraction of the muscle, the innominate artery was exposed as well as the bifurcation [5]. This exposure allowed for the placement of a side-biting clamp with an end-to-side anastomosis of an 8-mm graft to the innominate artery with tunneling of the graft out through the right neck and subsequent placement of the Impella 5.5 [5]. This approach, like ours, avoids partial sternotomy and gives excellent exposure to the innominate artery and proximal subclavian artery. Extension of the incision along the anterior border of the sternocleidomastoid may allow for easier placement of an adequately sized side-biting clamp on the innominate artery, which was not possible through our smaller sternal notch incision that did not extend along the sternocleidomastoid. This literature promotes the central placement of Impella in the pediatric population, and our paper proposes that this approach is also feasible in the adult population, as an alternative, if the axillary artery cannot be used for implantation.

Implicated differences between a suprasternal incision over the standard subclavicular incision include increased distance from axillary lymphatics as well as the brachial plexus, which may theoretically decrease the risk of potential morbidity related to the incision and exposure. Should the patient progress to require LVAD or heart transplant, a suprasternal incision that is contiguous with the sternotomy incision avoids a separate and second wound site at the subclavicular area, which can decrease the risk of wound complications by limiting the number of wounds. However, in the case of infection in the suprasternal approach, this would be a disadvantage if the patient requires reoperation or heart transplant through the same site. Lastly, this alternative approach of central cannulation may help avoid direct aortic Impella implantation through a partial sternotomy.

## Conclusion

The Impella 5.5 has traditionally been implanted via the right axillary artery and represents the most common approach for placement. In a subset of patients with inadequate axillary artery diameter or prohibitive atherosclerotic disease, we propose that the Impella 5.5 can be implanted into the proximal subclavian artery or possibly the distal innominate artery using a limited suprasternal incision, with or without supraclavicular extension, depending on the target vessel. This approach is a reasonable alternative and is advantageous in this patient population with few potential limitations; however, patients must be assessed on a case-by-case basis to determine if they are a candidate for this alternative approach.
