# Correction to: Suprasternal approach for insertion of Impella 5.5 into the proximal right subclavian artery

**DOI:** 10.1007/s12055-024-01765-y

**Published:** 2024-06-07

**Authors:** Jay A. Patel, Zubair A. Hashmi

**Affiliations:** Department of Cardiothoracic Surgery, Pauley Heart Center, Virginia Commonwealth University, Richmond, VA USA

**Correction to: Indian Journal of Thoracic and Cardiovascular Surgery. 2024;40(3):400–403**

10.1007/s12055-024-01699-5

The statement for which we are submitting the erratum is the following:

"In our overall experience, we have resorted to this alternate approach very rarely, in a total of eight patients, while the large majority have been performed in the standard right axillary position. Of these eight patients, seven were performed for small vessel caliber (one of which was an aborted supraclavicular approach for inadequate proximal vessel caliber with variant carotid anatomy), and one was performed in the setting of a left-sided implantable cardioverter-defibrillator (ICD) that was re-sited to the right, thus necessitating a central approach to avoid a re-operative field."

Reason and Statement:

We want to clarify that this statement can potentially misrepresent the number of alternate approaches that have been performed at our current institution, which is not the case. The number in the original manuscript represents the number of alternate approaches that have been performed in our overall experience across all affiliated institutions. Overall, this manuscript strictly serves to function as a techniques paper to review the technical aspects of alternate approach Impella.

In our current institution, we have resorted to this alternate approach very rarely, in a total of only three patients, while the large majority have been approached via the standard right axillary position. In one of these three cases, the artery was still too small to accommodate the graft, which also had a variant carotid artery anatomy. We successfully were able to place the graft on the other two patients. One received an Impella successfully, while the other had an aortic root dissection and was placed on ecmo via this approach of subclavian graft.

